# Refinement and evaluation of an automated mass spectrometer for nitrogen isotope analysis by the Rittenberg technique

**DOI:** 10.1155/S1463924691000433

**Published:** 1991

**Authors:** R. L. Mulvaney, Y. P. Liu

**Affiliations:** Department of Agronomy University of Illinois Turner Hall 1102 South Goodwin Avenue Urbana Illinois 61801 USA

## Abstract

An apparatus designed to automatically perform hypobromite oxidations of ammonium salt samples for nitrogen isotope analyses with a mass spectrometer was modified to improve performance and reduce analysis time. As modified, reference N_2_ is admitted to the mass spectrometer between samples from a dedicated inlet manifold, for calibration at the same pressure as that of the preceding sample. Analyses can be performed on samples containing 10 μg to 1 mg of N (or more), at a rate of up to 350 samples/day. When operated with a double-collector mass spectrometer, the standard deviation at the natural abundance level (10 analyses, 50-150 μg N) was <0.0001 atom % ^15^N. Very little memory was observed when natural abundance samples (0.366 atom % ^15^N) were analysed. following samples containing 40 atom % ^15^N. Analyses in the range, 0.2 to 1 atom % ^15^N (50-150 μg N), were in good agreement with manual Rittenberg analyses (1 mg N) using a dual-inlet system, and precision was comparable. For enrichments of 2 to 20 atom % ^15^N, automated analyses were slightly lower than manual analyses, which was attributed to outgassing of N_2_ from the plastic microplate used to contain samples.


Journal of Automatic Chemistry, Vol. 13, No. 6 (November–Decmber 1991), pp. 273–280

### Refinement and evaluation of an automated mass spectrometer for nitrogen isotope analysis by the Rittenberg technique

**R. L. Mulvaney and Y. P. Liu**

*Department of Agronomy, University of Illinois, Turner Hall, 1102 South Goodwin Avenue, Urbana, Illinois 61801, USA*

*An apparatus designed to automatically perform hypobromite oxidations of ammonium salt samples for nitrogen isotope analyses with a mass spectrometer was modified to improve performance and reduce analysis time. As modified, reference $N_2$ is admitted to the mass spectrometer between samples from a dedicated inlet manifold, for calibration at the same pressure as that of the preceding sample. Analyses can be performed on samples containing 10 μg to 1 mg of N (or more), at a rate of up to 350 samples/day. When operated with a double-collector mass spectrometer, the standard deviation at the natural abundance level (10 analyses, 50–150 μg N) was <0·0001 atom % $^{15}N$. Very little memory was observed when natural abundance samples (0·366 atom % $^{15}N$) were analysed following samples containing 40 atom % $^{15}N$. Analyses in the range, 0·2 to 1 atom % $^{15}N$ (50–150 μg N), were in good agreement with manual Rittenberg analyses (1 mg N) using a dual-inlet system, and precision was comparable. For enrichments of 2 to 20 atom % $^{15}N$, automated analyses were slightly lower than manual analyses, which was attributed to outgassing of $N_2$ from the plastic microplate used to contain samples.*

### Introduction

Use of nitrogen-15 as an isotopic tracer has been stimulated by the automation of mass spectrometers for nitrogen isotope analysis. Automation may be accomplished by interfacing an automatic N/C analyser (ANCA) to an isotope ratio mass spectrometer [1–4]; the combination is commonly referred to as ANCA-MS. During analyses, N in the sample is converted to $N_2$ and N oxides by flash combustion of a Sn sample container (≃1700 °C) in the presence of CuO and a catalyst (usually $Cr_2O_3$). Reduction of the N oxides to $N_2$ occurs as the combustion products are swept over Cu at 600 °C. The $N_2$ is purified by gas chromatography, and a small fraction (≃1%) of the effluent is admitted to the mass spectrometer for measurement of the ion currents at *m/z* 28, 29 and 30, from which both total N and $^{15}N$ are determined.

Nitrogen isotope analyses can also be performed by the Rittenberg technique, which utilizes alkaline hypobromite to oxidize ammonium ($NH_4^+$) to $N_2$ in the absence of air. This technique, named after its originator, has been the method of choice for manual N isotope analyses for more than 50 years. Conversion of N in the sample to $NH_4^+$ is commonly carried out by the Kjeldahl method [5], which involves digestion with concentrated $H_2SO_4$ to convert organic forms of N to $NH_4^+$-N, followed by steam distillation of the digest with alkali.

A project to automate Rittenberg analyses of $NH_4^+$ salt samples was initiated in 1978 by McInteer and Montoya at the Los Alamos National Laboratory. The result was an automated mass spectrometer that could perform isotope-ratio analyses on microgram quantities of N, at a rate of up to several hundred samples per day [6]. For analyses with this instrument, dried $NH_4^+$ samples were placed in miniature plastic vials, which were held in a sample tray (137 vials/tray). The tray was moved with a modified *x–y* plotter to sequentially position the vials beneath a pneumatically actuated reaction head designed to make a gas-tight seal with a single vial. Air was purged from the vial with Freon, a small amount of hypobromite was introduced, and the $N_2$ generated was admitted to a vacuum manifold equipped with a liquid $N_2$ trap for removal of Freon. Pressure in the manifold was measured by a pressure transducer and reduced, if necessary, by momentarily opening a valve to vacuum. The $N_2$ was then admitted to the mass spectrometer for isotope-ratio analysis, followed by removal of the residual $N_2$ and heating of the liquid $N_2$ trap to remove Freon. A programmable calculator was utilized for data acquisition and control.

To test the automated mass spectrometer developed at the Los Alamos National Laboratory, thousands of analyses were performed for scientists engaged in N isotope research. Eventually, a private business was established to continue this service (Isotope Services, Los Alamos, New Mexico), using the same type of automated mass spectrometer. The second instrument incorporated several refinements, including the use of disposable microplates to contain samples and the capability for multiple loading of plates [7].

A commercial system based on the design of McInteer and Montoya [6] was recently developed for automation of a mass spectrometer in the authors' laboratory [8]. Experience gained in the operation of this system for analyses of more than 30 000 samples has led to several modifications which improve performance. The major modifications are described in this article together with an evaluation of analytical performance.

### Experimental

### *Hardware*

The automated Rittenberg apparatus (ARA) used is a prototype unit that was developed in co-operation with Measurement and Analysis Systems (MAAS, formerly Nuclide Corp.), Bellefonte, Pennsylvania, USA, for

0142–0453/91 $3.00 © 1991 Taylor & Francis Ltd.

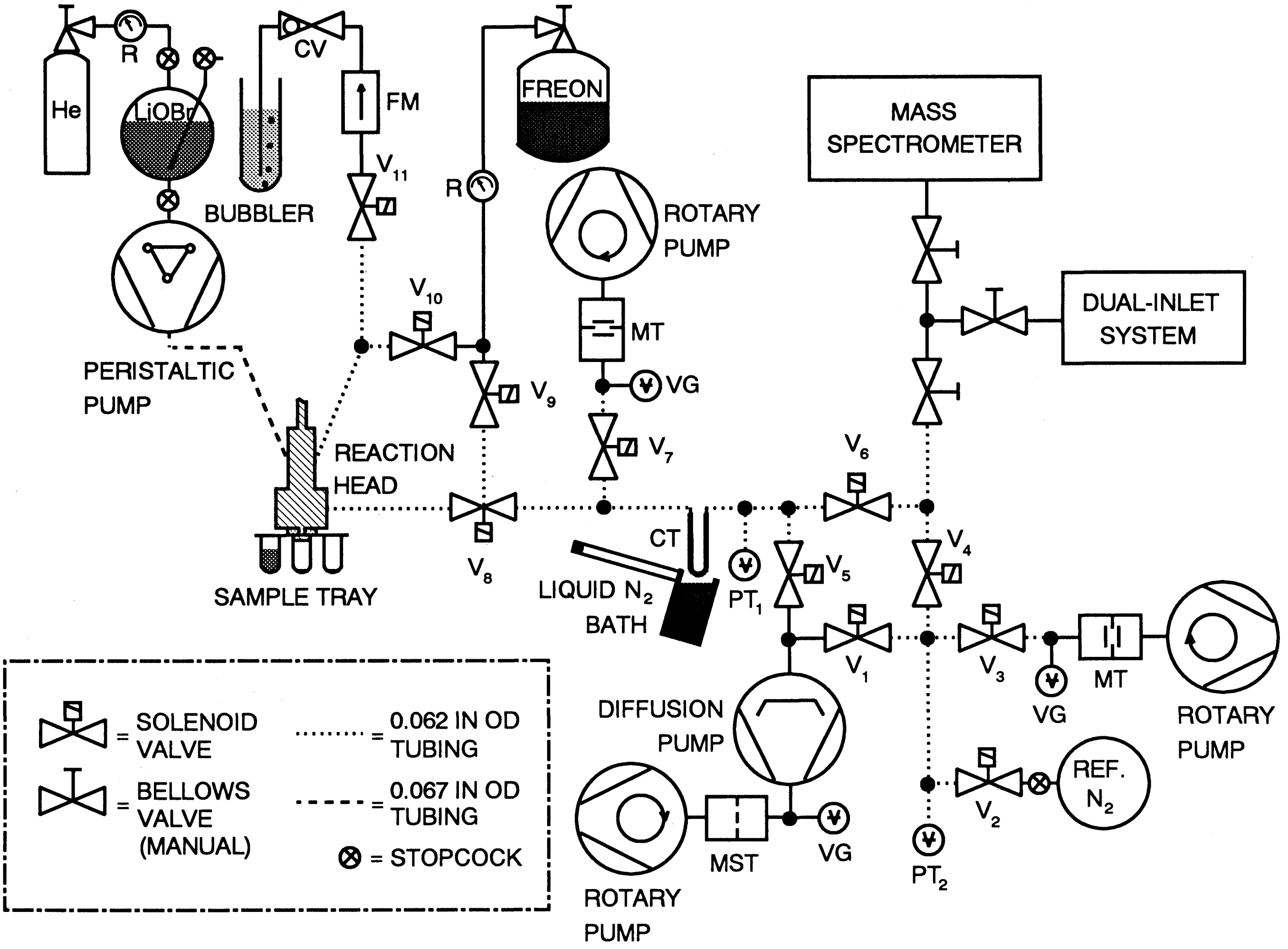

*Figure 1. Schematic diagram illustrating automated Rittenberg apparatus (ARA). CT, cold trap (resistance-heated); CV, check valve; FM, flowmeter (Omega Model FMA-5707); MST, molecular sieve trap; MT, Micromaze trap (Kurt J. Lesker Co., Clairton, Pennsylvania); PT, pressure transducer (MAAS Model TD101); VG, vacuum gauge (thermocouple); R, regulator. The ARA and a dual-inlet system connect to the mass spectrometer via manually operated bellows valves (Nupro Model SS-4H).*

operation with a double-collector mass spectrometer (Nuclide/MAAS Model 3-60-RMS) [8]. The mass spectrometer is also equipped with a conventional dual-inlet system. This system consists of a vacuum manifold with two variable volume metal bellows (10—300 ml), matched viscous leaks and a set of four changeover valves (Nupro Model SS-4BK-1C) to sequentially admit sample and reference gases to the mass spectrometer and to a waste pump (an 11 l/s Perkin–Elmer Ultek ion pump). Connection of the ARA and dual-inlet system to the mass spectrometer is made via a stainless-steel tee with three bellows valves (Nupro Model SS-4H), which allows either dual-inlet or automated operation to be conveniently selected.

The ARA operates under computer control to liberate $N_2$ from a preselected number of $NH_4^+$ salt samples, regulate the pressure of $N_2$ liberated, admit the $N_2$ to the

*Table 1. Expressions used to calculate atom % $^{15}N$ for $NH_4^+$ samples.*

| Nominal atom % $^{15}$N | Ratio measured† ($r$) | Expression for calculating atom % $^{15}$N‡ |
|---|---|---|
| <5 | $^{29}N_2/(^{28}N_2 + {}^{30}N_2)$ | $100\left(\dfrac{-2-2\Delta r-2K_1+(4-4\Delta r^2-4K_1{}^2-8K_1\Delta r)^{1/2}}{-4-4\Delta r-4K_1}\right)$ |
| ≥5 | $^{30}N_2/(^{28}N_2 + {}^{29}N_2)$ | $100\,[(\Delta r + K_2)/(1 + \Delta r + K_2)]^{1/2}$ |

† Ratios are corrected for the resistances associated with the electrometer heads.
‡ $\Delta r = r_{\text{sample}} - r_{\text{reference}}$. $K_1 \equiv 2C(1-C)/[C^2 + (1-C)^2]$. $K_2 \equiv C^2/[(1-C)^2 + 2C(1-C)]$. $C$ is the atom fraction of $^{15}N$ in the reference $N_2$ (0·003663 for ambient air).

mass spectrometer for isotope-ratio analysis and evacuate residual $N_2$ following the analysis. Several modifications were made in the original design [8] to reduce analysis time, improve performance and increase operational reliability. Figure 1 illustrates the modified plumbing arrangement schematically.

As originally designed [8], the ARA was equipped with a single inlet manifold, from which either sample or reference $N_2$ was admitted to the mass spectrometer. To increase throughput capacity, multiple samples could be analysed between calibrations; however, higher precision was achieved by analysing reference $N_2$ after every sample. Ideally, the latter approach is carried out using separate inlet manifolds for sample and reference $N_2$, so that calibration can be accomplished as soon as possible following isotope-ratio analysis of the sample, without prolonging the operational routine. This is achieved with the design illustrated by figure 1. The manifold for reference $N_2$ consists of valves 1–4 (figure 1) connected via 1/16th in o.d. stainless-steel tubing. The sample manifold is defined by valves 5–8. Both manifolds are equipped with a pressure transducer (MAAS Model TD101) and a two-stage rotary pump (Alcatel Model 2002B) with foreline trap and thermocouple vacuum measurement, utilized in regulating inlet pressure prior to isotope-ratio analysis. A three-stage oil diffusion pump backed by a rotary pump allows rapid evacuation of residual $N_2$ from either manifold following the analysis.

An important consideration in the design of the ARA is the internal volume of the inlet manifold, which must be minimized to obtain sufficient pressure from microgram quantities of $N_2$ for analyses with the mass spectrometer [6,7]. To meet this requirement, the original design utilized 1/16th in o.d. tubing and tube fittings and miniature solenoid valves [8]. Subsequent work showed that a further increase in pressure could be attained by reducing the internal volume of the sample drawback valve (valve 8 in figure 1), used to introduce Freon during purging of the sample well and admit $N_2$ to the sample manifold following hypobromite oxidation of the $NH_4^+$ salt sample. The reduction in volume was achieved by replacing the body and plunger of the original three-way valve (Honeywell-Skinner Model B13ADK1150), which had 1/8th in NPT fittings, with those from a two-way valve (Honeywell-Skinner Model B2DA9400) having 1/16th in PTF fittings. The new plunger, like the original, was crossdrilled to ensure complete purging of air or $N_2$ from a previous sample. The transducer used to measure pressure in the sample manifold ($PT_1$ in figure 1), originally mounted to the body of the sample drawback valve, was relocated on the other side of the cold trap. In this position, the transducer is protected against corrosion in the event that hypobromite is admitted to the manifold during drawback (i.e. due to malfunction).

As specified previously [8], LiOBr is used to oxidize $NH_4^+$-N to $N_2$. The LiOBr is purged with He to exclude any air. It is stored in a sealed bulb under a He atmosphere during use. Experience revealed the need to maintain a constant pressure of He in the bulb as the volume of LiOBr decreased during operation, so He is now supplied at 1–4 psig via the stopcock used as a vent in purging (figure 1). To reduce contamination of the LiOBr by air during delivery to the reaction head, the Tygon pump tubing originally used with the peristaltic pump was replaced with Norprene tubing (0·125 in o.d., 0·031 in i.d.).

*Table 2. Comparison of Freon-12 and Freon-22 as purge gases.*†

| Type of Freon‡ | Atom % $^{15}$N determined ($N$ = 10) Range | Mean | SD |
|---|---|---|---|
| 12 | 0·5022–0·5026 | 0·50241 | 0·00013 |
| 22 | 0·5022–0·5026 | 0·50236 | 0·00014 |

† Analyses were performed on 50 μg of N as $(NH_4)_2SO_4$ (nominal $^{15}$N content = 0·5 atom % $^{15}$N).
‡ Freon was purified before use as described by Mulvaney *et al.* [8].

*Table 3. Effect of Freon purge time on analytical performance.*†

| Purge time (s) | Atom % $^{15}$N determined ($N$ = 10) Range | Mean | SD |
|---|---|---|---|
| 5 | 0·4927–0·4982 | 0·49590 | 0·00168 |
| 10 | 0·5002–0·5007 | 0·50048 | 0·00016 |
| 15 | 0·5005–0·5008 | 0·50068 | 0·00009 |
| 30 | 0·5011–0·5015 | 0·50131 | 0·00010 |
| 60 | 0·5018–0·5021 | 0·50193 | 0·00011 |
| 90 | 0·5019–0·5022 | 0·50214 | 0·00010 |
| 180 | 0·5021–0·5025 | 0·50238 | 0·00013 |
| 360 | 0·5024–0·5030 | 0·50274 | 0·00024 |
| 15‡ | 0·5021–0·5026 | 0·50242 | 0·00015 |
| 30‡ | 0·5024–0·5026 | 0·50249 | 0·00008 |
| 45‡ | 0·5024–0·5027 | 0·50254 | 0·00009 |
| 60‡ | 0·5023–0·5027 | 0·50254 | 0·00012 |

† Analyses were performed on 50 μg of N as $(NH_4)_2SO_4$ (nominal $^{15}$N content = 0·5 atom % $^{15}$N).
‡ Purging was performed for the period specified following an initial purge for 10 s and a 4-min period during which the sample well was pressurized with Freon (3–4 psig).

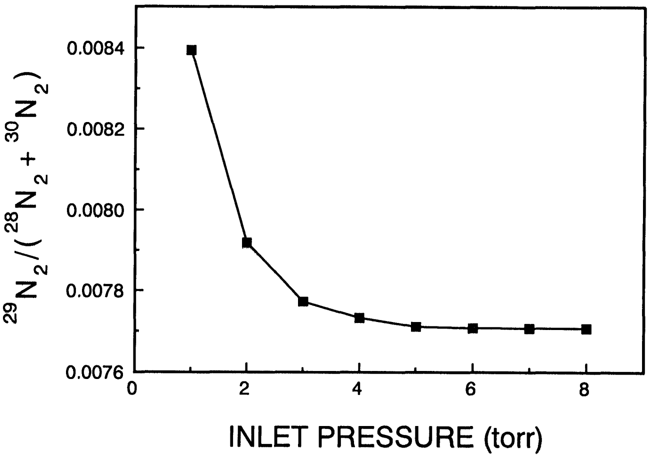

*Figure 2. Ratio data for different inlet pressures of reference $N_2$.*

*Table 4. Comparison of analytical performance with and without the use of regression for pressure regulation of reference $N_2$.†*

| Inlet pressure (torr) | Ion current, $m/z$ 28 + 30 (range for 10 analyses, pA) | | Atom % $^{15}N$ determined ($N$ = 10) | | |
|---|---|---|---|---|---|
| | Sample | Reference | Range | Mean | SD |
| | | | *Without regression* | | |
| 1·0 | 0·9–1·1 | 3·5–4·5 | 0·5180–0·5334 | 0·52950 | 0·00669 |
| 2·0 | 3·6–4·3 | 9·8–10·8 | 0·5071–0·5186 | 0·50983 | 0·00324 |
| 3·0 | 9·7–10·4 | 21·8–22·8 | 0·5058–0·5068 | 0·50637 | 0·00027 |
| 4·0 | 17·3–19·8 | 35·6–38·2 | 0·5047–0·5055 | 0·50527 | 0·00025 |
| 5·0 | 30·3–32·7 | 51·8–57·0 | 0·5044–0·5051 | 0·50466 | 0·00024 |
| 6·0 | 45·9–49·1 | 72·0–75·2 | 0·5033–0·5039 | 0·50368 | 0·00022 |
| 7·0 | 60·4–66·3 | 89·0–93·3 | 0·5029–0·5034 | 0·50318 | 0·00015 |
| 8·0 | 85·5–91·2 | 115·0–122·5 | 0·5025–0·5028 | 0·50268 | 0·00010 |
| | | | *With regression* | | |
| 1·0 | 1·0–1·1 | 1·0–1·5 | 0·4989–0·5140 | 0·50541 | 0·00574 |
| 2·0 | 4·1–4·4 | 3·7–4·3 | 0·4998–0·5021 | 0·50140 | 0·00065 |
| 3·0 | 9·7–11·2 | 10·2–11·3 | 0·5015–0·5021 | 0·50186 | 0·00022 |
| 4·0 | 18·5–21·4 | 15·4–18·2 | 0·5015–0·5022 | 0·50176 | 0·00027 |
| 5·0 | 29·2–36·5 | 26·2–34·7 | 0·5023–0·5028 | 0·50250 | 0·00013 |
| 6·0 | 47·6–51·1 | 45·0–51·6 | 0·5022–0·5027 | 0·50242 | 0·00014 |
| 7·0 | 61·2–67·5 | 59·7–68·9 | 0·5022–0·5027 | 0·50248 | 0·00017 |
| 8·0 | 86·2–95·5 | 90·5–100·9 | 0·5019–0·5023 | 0·50210 | 0·00016 |

† Analyses were performed on 150 μg of N as $(NH_4)_2SO_4$ (nominal $^{15}N$ content = 0·5 atom % $^{15}N$).

Freon admitted to the sample inlet manifold during drawback is immediately frozen out in a U-type cold trap, which is immersed in liquid $N_2$ by raising an Al arm that supports a Dewar (figure 1). The supply of liquid $N_2$ to the Dewar is regulated by a liquid $N_2$ level controller (not shown in figure 1). The cold trap is formed from tubing of sufficient diameter to preclude blockage upon freeze-out of Freon, but blockage can occur in the smaller diameter tubing to which the cold trap connects if the Dewar fills during drawback, in which case the analysis must be aborted. To limit the period for filling of liquid $N_2$, a relay was installed between the liquid $N_2$ level controller and the solenoid filling valve. This relay is also used to prevent filling of liquid $N_2$ while the ARA is in standby status.

*Table 5. Magnitude of memory in analysis of samples differing in $^{15}N$ content.†*

| Atom % $^{15}N$ of labelled sample | Atom % $^{15}N$ determined for subsequent unlabelled samples | | |
|---|---|---|---|
| | Sample no. 1 | Sample no. 2 | Sample no. 10 |
| 0·5 | 0·3651 | 0·3653 | 0·3650 |
| 1·0 | 0·3651 | 0·3649 | 0·3650 |
| 2·0 | 0·3652 | 0·3650 | 0·3653 |
| 5·0 | 0·3655 | 0·3652 | 0·3655 |
| 10·0 | 0·3659 | 0·3655 | 0·3654 |
| 20·0 | 0·3662 | 0·3653 | 0·3654 |
| 30·0 | 0·3665 | 0·3657 | 0·3651 |
| 40·0 | 0·3666 | 0·3656 | 0·3655 |

† Analyses were performed on 10 consecutive unlabelled $(NH_4)_2SO_4$ samples (50 μg N, nominal $^{15}N$ content = 0·366 atom % $^{15}N$) following a sample containing 0·5, 1, 2, 5, 10, 20, 30 or 40 atom % $^{15}N$.

The automated mass spectrometer is capable of unattended operation for several days, so the original design [8] included provision to monitor various phases of operation and to abort a run in the event of system failure. Additional protection was achieved by installing a MetraByte PDISO-8 interface board in the IBM PC microcomputer used for system control. The PDISO-8 board is equipped with eight electro-mechanical relay outputs and eight isolated inputs for control and sensing applications. As installed, it monitors the status of the liquid $N_2$ level controller, the cold trap heater and a turbomolecular pump on the mass spectrometer [8], and controls the relay that limits filling of liquid $N_2$. An electronic flowmeter (figure 1) monitors Freon flow during purging of the sample well for the purpose of detecting insufficient flow, which may be caused by low Freon pressure, plugging of the drawback or exhaust line or improper alignment of the reaction head with the sample well. The flowmeter (Omega Model FMA-5707) produces an analog output, utilized by the computer via the integrating ratiometer (MAAS/Nuclide Model IR-6). A polyethylene check valve connected to the outlet from the flowmeter (figure 1) prevents water in the bubbler from entering the inlet manifold if the exhaust line is exposed to vacuum as the result of malfunction.

### *Software*

The original software was modified to improve user convenience, extend hardware support and enable operation via a more efficient routine.

For convenience, analyses may begin with any well in a tray, and trays with partially filled or empty rows can be processed. Up to eight trays can be processed in a single run, and, if necessary a new set of operating parameters

*Table 6. Comparison of $^{15}N$ analyses using the mass spectrometer with a manual dual-inlet system or the ARA.*

| Nominal atom % $^{15}N$ of $(NH_4)_2SO_4$† | Type of analysis | $NH_4^+$-N submitted for analysis (μg) | Atom % $^{15}N$ determined ($N = 10$) | | |
|---|---|---|---|---|---|
| | | | Range | Mean | SD |
| 0·2 | Dual-inlet | 1000 | 0·2053–0·2058 | 0·20554 | 0·00018 |
| | ARA | 150 | 0·2043–0·2049 | 0·20455 | 0·00016 |
| | | 100 | 0·2041–0·2049 | 0·20442 | 0·00023 |
| | | 50 | 0·2039–0·2048 | 0·20440 | 0·00031 |
| | | 20 | 0·2075–0·2098 | 0·20803 | 0·00065 |
| | | 10 | 0·2127–0·2163 | 0·21442 | 0·00126 |
| NA | Dual-inlet | 1000 | 0·3656–0·3659 | 0·36583 | 0·00007 |
| | ARA | 150 | 0·3650–0·3651 | 0·36507 | 0·00006 |
| | | 100 | 0·3650–0·3653 | 0·36518 | 0·00008 |
| | | 50 | 0·3656–0·3658 | 0·36568 | 0·00008 |
| | | 20 | 0·3663–0·3668 | 0·36661 | 0·00015 |
| | | 10 | 0·3669–0·3721 | 0·36822 | 0·00159 |
| 0·5 | Dual-inlet | 1000 | 0·5025–0·5026 | 0·50256 | 0·00006 |
| | ARA | 1000 | 0·5003–0·5016 | 0·50104 | 0·00040 |
| | | 500 | 0·5013–0·5019 | 0·50165 | 0·00023 |
| | | 250 | 0·5022–0·5025 | 0·50237 | 0·00011 |
| | | 150 | 0·5023–0·5027 | 0·50249 | 0·00012 |
| | | 100 | 0·5024–0·5032 | 0·50280 | 0·00023 |
| | | 50 | 0·5022–0·5026 | 0·50236 | 0·00014 |
| | | 20 | 0·5022–0·5030 | 0·50251 | 0·00030 |
| | | 10 | 0·4933–0·5000 | 0·49683 | 0·00208 |
| 1·0 | Dual-inlet | 1000 | 1·038–1·039 | 1·0386 | 0·0005 |
| | ARA | 150 | 1·035–1·038 | 1·0367 | 0·0007 |
| | | 100 | 1·035–1·039 | 1·0378 | 0·0012 |
| | | 50 | 1·038–1·040 | 1·0389 | 0·0007 |
| | | 20 | 1·026–1·029 | 1·0277 | 0·0013 |
| | | 10 | 1·001–1·013 | 1·0081 | 0·0046 |
| 2·0 | Dual-inlet | 1000 | 2·077–2·079 | 2·0784 | 0·0006 |
| | ARA | 150 | 2·072–2·075 | 2·0734 | 0·0008 |
| | | 100 | 2·073–2·079 | 2·0760 | 0·0022 |
| | | 50 | 2·078–2·083 | 2·0798 | 0·0020 |
| | | 20 | 2·042–2·057 | 2·0496 | 0·0040 |
| | | 10 | 1·956–1·994 | 1·9716 | 0·0103 |
| 5·0 | Dual-inlet | 1000 | 5·099–5·104 | 5·1032 | 0·0017 |
| | ARA | 150 | 5·091–5·096 | 5·0938 | 0·0015 |
| | | 100 | 5·085–5·092 | 5·0887 | 0·0024 |
| | | 50 | 5·063–5·069 | 5·0656 | 0·0019 |
| | | 20 | 4·972–4·982 | 4·9768 | 0·0034 |
| | | 10 | 4·703–4·822 | 4·7587 | 0·0451 |
| 10·0 | Dual-inlet | 1000 | 10·032–10·041 | 10·0363 | 0·0029 |
| | ARA | 150 | 9·911–9·921 | 9·9166 | 0·0044 |
| | | 100 | 9·905–9·919 | 9·9123 | 0·0041 |
| | | 50 | 9·886–9·900 | 9·8936 | 0·0042 |
| | | 20 | 9·793–9·825 | 9·8104 | 0·0099 |
| | | 10 | 9·473–9·629 | 9·5849 | 0·0601 |
| 20·0 | Dual-inlet | 1000 | 20·415–20·434 | 20·4228 | 0·0062 |
| | ARA | 150 | 20·201–20·230 | 20·2195 | 0·0081 |
| | | 100 | 20·178–20·209 | 20·1918 | 0·0088 |
| | | 50 | 20·136–20·166 | 20·1517 | 0·0079 |
| | | 20 | 19·898–19·951 | 19·9308 | 0·0166 |
| | | 10 | 19·426–19·702 | 19·5773 | 0·0972 |

† NA = natural abundance (0·366 atom % $^{15}N$).

can be retrieved from disk storage for each tray, or for each row within a tray. Moreover, the report may be written to a floppy disk as well as printed. This generates an ASCII file that can be retrieved to print additional copies of the report, or be imported into spreadsheet or word-processing software.

The software supports sensing and control functions via the PDISO-8 board, and enhances the control capabilities of the IEEE-488 interface utilized for I/O connection to the integrating ratiometer and valve interface [8]. For example, ion accelerating voltage is automatically controlled such that the measured ratio is $^{29}N_2/(^{28}N_2$ +

$^{30}N_2$) for samples containing <5 atom % $^{15}N$, and $^{30}N_2/(^{28}N_2 + {}^{29}N_2)$ for higher enrichments. In all cases, ratio measurements are performed only after voltage data have been collected to check for numerator overflow. If overflow occurs, inlet pressure is reduced until the condition is no longer detected, and the ratio analysis is then performed.

*Operation*

Analyses with the modified ARA follow the routine listed below (numbering of valves follows figure 1):

(1) In standby status, valves 1 and 5 are open to evacuate the sample and reference inlet manifolds. All other valves are closed.

(2) Upon receiving keyboard input to begin a run, the *x–y* table loads a sample tray (loading can be done from a stack of up to eight trays) and moves it to position the starting well (96 wells/tray) beneath the reaction head.

(3) The reaction head drops, establishing a gas-tight seal with the well.

(4) The sample well is purged with Freon by opening valves 9 and 11. Initial purging is for 10 s, during which the flow rate is measured, and valve 10 is opened briefly to eliminate air from the line that connects this valve to the exhaust line. Valves 9 and 11 are then closed, leaving the sample well under a positive pressure of Freon to promote exchange with air adsorbed on the plastic surface. After approximately 4 min, valves 9 and 11 are opened for 10–60 s to remove residual air.

(5) The peristaltic pump is operated for 5–15 s, introducing $\simeq$0·1 ml of LiOBr into the sample well for conversion of $NH_4^+$-N to $N_2$.

(6) The liquid $N_2$ bath is raised. Filling of liquid $N_2$ is disabled. A delay of 15–30 s is provided to ensure complete freezing of the cold trap and complete oxidation of $NH_4^+$ by LiOBr.

(7) Valve 5 is closed to isolate the sample inlet manifold.

(8) Freon and sample $N_2$ are admitted to the sample inlet manifold by opening valve 8 for a few milliseconds (the sample drawback time). Freon is frozen out in the cold trap.

(9) The reaction head is raised after momentarily opening valve 9 to pressurize the drawback line (this eliminates a partial vacuum from drawback that would otherwise lead to the entry of air into the drawback line and enhance jarring of the tray as the head is raised) and then operating the peristaltic pump for a few seconds to eliminate any voids from the reagent line. The head is cleaned by positioning a polypropylene box with the *x–y* table, lowering the head onto paper towels in the box, opening valves 9 and 10 for a few seconds and then raising the head.

(10) The sample tray is moved with the *x–y* table to position the next well beneath the reaction head.

(11) The head is lowered, and purging is performed for 10 s as described in step 4. Valves 9 and 11 are then closed to maintain a Freon atmosphere in the sample well.

(12) Filling of liquid $N_2$ is enabled.

(13) Pressure in the sample manifold is measured with the pressure transducer ($PT_1$). If the measured pressure ($P_m$) is <1 torr, the analysis is aborted, and clean-up of the cold trap occurs. If $P_m$ exceeds a specified inlet regulation pressure ($P_r$ = 1–10 torr), pumping is accomplished by opening valve 7. Initial pumping may be continuous ($P_m > 1{\cdot}25\ P_r$), in which case the pressure is also monitored continuously; final pumping ($P_r < P_m \leq 1{\cdot}25P_r$) is intermittent, with valve 7 being opened momentarily after a 3-s delay for pressure measurement. The final sample pressure ($P_s$), obtained with ($P_s \simeq P_r$) or without ($P_s \leq P_r$) regulation, is stored in memory.

(14) Valve 6 is opened to admit sample $N_2$ to the mass spectrometer.

(15) A delay of 30–60 s is provided to allow equilibration of the ion source. Immediately after beginning this delay, valve 1 is closed to isolate the reference inlet manifold, and valve 2 is opened for a few milliseconds to admit reference $N_2$ from a 1-l bulb. Pressure in the reference manifold is then measured with the pressure transducer ($PT_2$). If the measured pressure ($P_m'$) is $<P_s$, drawback via valve 2 is repeated. If $P_m'$ is $>1{\cdot}25P_s$, valve 3 is opened, and $P_m'$ is continuously updated. Valve 3 is closed when $P_m'$ is $\leq P_s$, or upon completing the delay period.

(16) Ratio data are collected for sample $N_2$ after checking for numerator overflow. If preliminary measurements of $^{29}N_2/(^{28}N_2 + {}^{30}N_2)$ indicate a $^{15}N$ content >5 atom %, the ion accelerating voltage is decreased to measure $^{30}N_2/(^{28}N_2 + {}^{29}N_2)$.

(17) Valve 5 is opened to evacuate residual $N_2$ from the sample manifold. A delay of 15–60 s ensures complete evacuation. During this period, final pumping of reference $N_2$ is carried out by intermittently opening valve 3 until $P_m'$ is $\leq P_s$.

(18) Valve 6 is closed to isolate the sample inlet manifold from the mass spectrometer, and valve 4 is opened to admit reference $N_2$.

(19) A delay of 30–60 s allows equilibration of the ion source. At the beginning of this delay, valve 5 is closed, valve 7 is opened, the liquid $N_2$ bath is lowered, and heating of the cold trap (to remove Freon) is initiated. Filling of liquid $N_2$ is disabled.

(20) Ratio data are collected for reference $N_2$ after checking for numerator overflow. As data collection begins, valves 9 and 11 are opened to complete purging of the next sample well. Purging is terminated (i.e by closing valves 9 and 11) after 10–60 s, which may occur during or after data collection. Heating of the cold trap is concluded during data collection.

(21) Upon completing the analysis of reference $N_2$, atom % $^{15}N$ is calculated for the sample (see table 1), and a report is printed. Valve 1 is opened to evacuate the reference manifold. Evacuation of the sample manifold is completed by closing valve 7 and opening valve 5. The routine continues via step 5.

### Evaluation

The automated mass spectrometer is designed to process large numbers of samples without operator intervention. Up to 768 samples can be accommodated in a single loading, and operation can continue 24 h per day. With samples containing at least 50 μg of N, throughput capacity is 300–350 samples per day. Capacity is reduced with smaller samples because of slower integration with the ratiometer and prolonged pumping to regulate the pressure of reference $N_2$. However, throughput still exceeds 200 samples per day with 20 μg of N.

When purified to remove $N_2$ [7,8], Freon-12 ($CCl_2F_2$) is an excellent purge gas for the ARA. However, international concern over depletion of stratospheric ozone by chlorofluorocarbon (CFC) refrigerants has led to recent legislation that progressively restricts their consumption during the 1990s, and bans all further production as of 2000 [9]. This ban does not apply to hydrochlorofluorocarbon (HCFC) refrigerants, which have much shorter atmospheric lifetimes than CFCs, and, hence, lower potential for depletion of ozone [9]. The latter group includes Freon-22 ($CHClF_2$), which is commonly used in air-conditioning systems. Data in table 2 show that Freon-22 is a satisfactory purge gas for the ARA. As with Freon-12, purification is necessary to remove $N_2$. This can be accomplished using the apparatus described previously [8]; however, a higher pressure is generated with Freon-22 than with Freon-12 (the pressure during purification of Freon-22 will reach approximately 200 psig, as opposed to 130–140 psig for Freon-12), and the bubbler valve (used to exhaust Freon contaminated with $N_2$) must be heated to maintain a stable flow rate. Attempts to use $CO_2$ as a purge gas were unsuccessful due to formation of $Br_2$ upon reaction of LiOBr with $H_2CO_3$.

In a previous evaluation of analytical precision with the ARA [8], analyses of $^{15}N$-enriched $(NH_4)_2SO_4$ were found to decrease, and analyses of $^{15}N$-depleted $(NH_4)_2SO_4$ to increase, with decrease in sample size from 100 to 20 μg of N. These findings were attributed to isotopic contamination from atmospheric $NH_3$ [8]. Subsequent work showed that the major problem is the incomplete removal of air during the purging of the sample well with Freon for 16–24 s. A comparison of $^{15}N$ analyses using different purge times (table 3) indicated that several minutes are required for complete purging, presumably because of outgassing by the polystyrene sample well. A more practical alternative, utilized in operation of the modified ARA, is to carry out purging in two stages, separated by several minutes during which a Freon atmosphere is maintained in the sample well. The effectiveness of this technique is apparent from table 3, which includes data from analyses involving final purge times of 15 to 60 s.

An important consideration in the use of mass spectrometry to measure isotope ratios is that ratio measurements depend directly upon inlet pressure. This is illustrated by figure 2, which shows data obtained for reference $N_2$ when the ratio, $^{29}N_2/(^{28}N_2 + {}^{30}N_2)$, was measured using inlet pressures of 1 to 8 torr. In every case, the measured ratio was higher than the theoretical value (0·00735 for the natural abundance level of $^{15}N$). Analyses of sample $N_2$ are subject to the same error, which necessitates the use of reference $N_2$ so that a ratio difference can be measured. An accurate value of atom % $^{15}N$ can then be obtained for the sample, provided both analyses were performed at the same pressure of $N_2$. Calibrations with the ARA were originally carried out by regulating the same inlet pressure for sample and reference $N_2$ [8], but this was found to give a larger ion current at $m/z$ 28 + 30 (which is proportional to the pressure of $N_2$) for the reference $N_2$ than for the sample $N_2$, owing to a lower content of non-liquid $N_2$ condensable impurities, and the result was overestimation of atom % $^{15}N$. To avoid such difficulty, regression equations were developed that relate the inlet pressure of sample or reference $N_2$ to the ion current generated at $m/z$ 28 + 30. These equations are utilized to obtain the same ion current with sample and reference $N_2$. Their effect on analytical performance is apparent from table 4, which summarizes data obtained when analyses were performed at different inlet pressures with and without the use of regression for pressure regulation of reference $N_2$. Without regression, atom % $^{15}N$ was consistently overestimated, and serious error occurred at low inlet pressures. With regression, overestimation was largely eliminated, although precision was limited at 1 torr by the tolerance in pressure regulation (±5%), due to the marked effect of low inlet pressures on ratio measurements (see figure 2).

As noted previously, the drawback valve utilized in the ARA is equipped with a crossdrilled plunger to ensure that all traces of $N_2$ from a previous sample are removed during purging with Freon. Moreover, strong pumping is provided by a 175 l/s diffusion pump connected to the inlet manifolds, and by a 50 l/s turbomolecular pump connected to the ion source of the mass spectrometer [8]. The combination is so effective that memory is virtually nonexistent, even when a natural abundance sample is analysed following a sample that contains 40 atom % $^{15}N$ (see table 5).

To evaluate the accuracy and precision of analyses with the ARA, a comparison was made to manual Rittenberg analyses with the dual-inlet system, using $(NH_4)_2SO_4$ at eight different $^{15}N$ concentrations between 0·2 and 20 atom %. Sample size for the ARA usually ranged from 10 to 150 μg of N, but in one case the range was from 10 to 1000 μg of N. Manual analyses were performed on 1000 μg of $NH_4^+$-N, which was oxidized to $N_2$ in disposable glass vials [10]. Table 6 summarizes the data obtained.

Examination of table 6 reveals that the accuracy and precision of analyses with the ARA depend upon the amount of N in the sample and the $^{15}N$ content. Best results were achieved with 50–150 μg of N, and an $^{15}N$ concentration between 0·2 and 1 atom %. Accuracy and precision were sacrificed with smaller samples and/or higher $^{15}N$ enrichments, presumably due to isotopic dilution by trace amounts of natural abundance $N_2$ outgassed from the plastic sample tray. No difficulty was encountered in the analysis of samples containing 250–1000 μg of N; however, the values obtained for $^{15}N$-enriched $(NH_4)_2SO_4$ (0·5 atom % $^{15}N$) were somewhat lower than with 50–150 μg of N, which can be attributed to isotopic fractionation from incomplete oxidation of $NH_4^+$-N to $N_2$ (the 0·1 ml of LiOBr added is sufficient to oxidize approximately 200 μg of N).

### Acknowledgements

The work reported here was a part of Project ILLU-15-0392, Illinois Agricultural Experimental Station, College of Agriculture, University of Illinois, Urbana, Illinois, USA. Appreciation is expressed to C. L. Fohringer, Zetachron Corporation, for advice concerning several modifications that were made to the ARA.

### References

1. Otsuki, A., Ino, Y. and Fujii, T., *International Journal of Mass Spectrometry and Ion Physics,* **48** (1983), 343.
2. Preston, T. and Owens, N. J. P., *Analyst,* **108** (1983), 971.
3. Barrie, A. and Workman, C. T., *Spectroscopy International Journal,* **3** (1984), 439.
4. Marshall, R. B. and Whiteway, J. N., *Analyst,* **110** (1985), 867.
5. Bremner, J. M. and Mulvaney, C. S., in *Methods of Soil Analysis,* Part 2, 2nd edn, Eds Page, A. L. *et al.* (American Society of Agronomy, Madison, Wisconsin, 1982), 595.
6. McInteer, B. B. and Montoya, J. G., in *Recent Developments in Mass Spectrometry in Biochemistry, Medicine and Environmental Research,* Ed. Frigerio, A. (Elsevier, Amsterdam, 1981), 343.
7. McInteer, B. B., Montoya, J. G. and Stark, E. E., *Spectroscopy International Journal,* **3** (1984), 226.
8. Mulvaney, R. L., Fohringer, C. L., Bojan, V. J., Michlik, M. M. and Herzog, L. F., *Review of Scientific Instruments,* **61** (1990), 897.
9. Gilkey, H. T., *Heating Piping Air Conditioning,* **63**(4) (1991), 41.
10. Buresh, R. J., Austin, E. R. and Craswell, E. T., *Fertilizer Research,* **3** (1982), 37.

